# Substantia Nigra Abnormalities Provide New Insight on the Neural Mechanisms Underlying the Sleep-Arousal Phase Dysfunctions in Sudden Infant Death Syndrome

**DOI:** 10.1177/1759091420962695

**Published:** 2020-09-30

**Authors:** Anna M. Lavezzi, Riffat Mehboob, Graziella Alfonsi, Stefano Ferrero

**Affiliations:** 1 “Lino Rossi” Research Center for the Study and Prevention of Unexpected Perinatal Death and SIDS, Department of Biomedical, Surgical and Dental Sciences, University of Milan, Milan, Italy; 2 Faculty of Allied Health Sciences, University of Lahore, Lahore, Pakistan; 3 Division of Pathology, Fondazione IRCCS Ca' Granda, Ospedale Maggiore Policlinico, Milan, Italy; 4 Department of Biomedical, Surgical and Dental Sciences, University of Milan, Milan, Italy

**Keywords:** neuropathology, substantia nigra, brain stem, sudden infant death syndrome, tyrosine hydroxylase, neuromelanin

## Abstract

The purpose of this study was to research possible developmental alterations of the substantia nigra (SN) in sudden infant death syndrome (SIDS), a syndrome frequently attributed to arousal failure from sleep. Brain stems of 46 victims of sudden infant death, aged from 1 to about 7 months (4 to 30 postnatal weeks), were investigated. Twenty-six of these cases were diagnosed as SIDS, due to the lack of any pathological finding, while the remaining 20 cases in which the cause of death was determined at autopsy served as controls. Maternal smoking was reported in 77% of SIDS and 10% of controls. Histopathological examination of the SN was done on 5-µm-thick sections of caudal midbrain stained with both hematoxylin-eosin and Klüver-Barrera. Densitometry, immunohistochemistry and histochemistry were applied to highlight the neuronal concentration, the tyrosine hydroxylase (TH) expression, and the presence of neuromelanin (NM) in this structure. Hypoplasia of the pars compacta portion of the SN was observed in 69% of SIDS but never in controls; TH expression was significantly higher in controls than in SIDS; and NM was observed only in 4 infants of the control group but not in SIDS. A significant correlation was found between SIDS, hypoplasia/low neuronal density, low TH expression in the pars compacta, and maternal smoking. Because the SN pars compacta, being the major dopamine brain center, controls many functions, including the sleep-arousal phase, its alterations, especially concurrently with smoking exposure, may contribute to explain the pathogenesis of SIDS that occur in the great part of cases at awakening from sleep.

The “substantia nigra” (SN) is a large midbrain structure that lies dorsal to the cerebral peduncles extending from the rostral border of the pons to the subthalamic region. Although the SN looks like a continuous band on both the right- and left-hand side of transverse histological sections, it is actually composed of two parts with very different connections and functions: the “pars compacta” (SNpc), a dorsal stratum of closely spaced pigmented cells that contain dopamine (DA) and neuromelanin (NM; a product of the DA synthesis), and the “pars reticulata” (SNpr), a larger ventral region of widely scattered and mainly GABAergic cells (Aubert et al., 1997; Sonne et al., 2020).

The SNpr conveys output signals from the basal ganglia to many other brain structures which use gamma aminobutyric acid (GABA) as neurotransmitter (such as thalamus and superior colliculus), while the SNpc serves as an input to the basal ganglia circuit, supplying the striatum with DA (Freeman et al., 1991; Hoffman and Gerhardt, 1999; Ikeda et al., 2010).

Moreover, the SNpc dopaminergic neurons play an important role in controlling many brain functions, especially voluntary movement (Bissonette and Roesch, 2016). Therefore, this region is associated with the motor abnormalities commonly observed in patients with Parkinson’s disease (PD), in which the degeneration of dopaminergic cells of the SNpc represents the most consistent pathological finding (Brooks, 1997; Jankovic, 2008).

Several studies have reported that, apart from controlling motor functions, DA also regulates the sleep–wake cycle (Dzirasa et al., 2006; Lima et al., 2007; Monti and Monti, 2007). DA deficiency in the SNpc can therefore lead to sleep disturbances, such as excessive daytime sleepiness and sleep behavior disorder which are common in patients with PD (Raggi et al., 2013; Gjerstad et al., 2018).

Because arousal failure from sleep has been frequently involved in the pathogenetic mechanism of sudden infant death syndrome (SIDS; Hunt, 1989; Kahn et al., 2003; Kato et al., 2003), the aim of this study was to evaluate possible developmental defects of SN in a large group of infants who died suddenly and unexpectedly in the first months of life. In particular, using immunohistochemical and histochemical techniques, we aimed to examine the cytoarchitecture of the SN in these cases and the expression of tyrosine hydroxylase (TH), which is an essential enzyme for the DA synthesis (Daubner et al., 2011), as well as NM, which is known to be a specific marker of dopaminergic neurons (Zecca et al., 2001). An ultimate goal was to provide a possible explanation as to how failures in the SN region can culminate in sudden death by SIDS.

## Methods

We investigated the structure of the SN and the expression of both TH and NM in its neurons in a cohort of 46 victims of sudden infant death, 26 males and 20 females, aged between 4 to 30 postnatal weeks (approximately 1–7 months), sent to the Lino Rossi Research Center in a 5-year period. Nine of these subjects were born premature (27 to 32 gestational weeks). The postmortem examination of the 46 cases was performed within 24 h from the death and in accordance with the guidelines provided by the Italian law n.31/2006 “*Regulations for Diagnostic Post Mortem Investigation in Victims of sudden Infant Death Syndrome (SIDS) and Unexpected Fetal Death*” (Available from: http://users.unimi.it/centrolinorossi/files/gazz_ufficiale.pdf).

### Ethics Approval

The institutional review board approval is not required for this study because it complies with the requirements of the Italian law n.31/2006. This law states that all victims of unexpected perinatal death must be subjected to extensive autopsy investigations, according to appropriate consistent guidelines. Furthermore, the Lino Rossi Research Center of the Milan University is the national referral center for the application of this law and the seat of the Data Bank for the collection of all data relating to cases of sudden fetal and infant death, as established by Article 3 of the aforementioned law. Anyway, the parents of all the infants included in the study provided written informed consent to autopsy, related researches, and publication of the results.

After the anatomopathological examination, 26 infant deaths were classified as “SIDS,” due to the lack, even with advanced diagnostics, of pathological findings, including any type of infection. Five of these victims were born premature and precisely 3 extremely preterm (less than 28 weeks) and 2 very preterm (28–32 gestational weeks). A precise cause of death was formulated at autopsy for the remaining 20 cases, 4 of which were born preterm (28–31 gestational weeks). As they shared certain sociodemographic characteristics (gender, ethnicity, and age) at the time of death with the SIDS victims, they were used as “controls.” For these 20 cases, the diagnoses were as follows: congenital heart disease (9 cases), severe bronchopneumonia (4 cases), pulmonary dysplasia (3 cases), myocarditis (2 cases), malaria (1 case), and pericarditis (1 case).

Before performing the autopsies, a complete clinical history, including information related to death circumstances, was collected for each victim.

### Child Family Information About Risk Factors for SIDS

None of the parents used illicit drugs or abused alcohol. The pregnant mothers generally did not use drugs such as antidepressants, anxiolytics, and antibiotics. Only a mother of a newborn who died suddenly from a congenital heart disease and therefore belonging to the control group declared that she had taken antipsychotic drugs throughout her pregnancy. No pathology has been reported in mothers before, during, and after pregnancy, including iron-deficiency anemia and diabetes. The mothers in particular were asked to complete a questionnaire concerning their smoking habits. Moreover, the guidelines of the Lino Rossi Research Center foresee the removal of a lock of the victims’ hair to test for xenobiotics and in particular cotinine, the main metabolite of nicotine, characterized by a long elimination half-life, using the gas chromatography with mass spectrometry.

Although it is difficult to determine an absolute cutoff concentration, more than 0.2 ng of cotinine per milligram of hair can be used in discriminating between exposed and unexposed infants (Florescu et al., 2007). The application of this test was aimed especially at verifying the negative assertions of the mothers, being aware that the retrospective assessment of maternal smoking habit, if performed after the fatal event, is sometimes unreliable because of guilt feelings (Connor Gorber et al., 2009). Twenty mothers of the 26 SIDS victims (77%), including 4 mothers who initially denied their smoking habit but with a positive cotinine test performed on the son’s hair, admitted to being active smokers (3 to 10 cigarettes a day since before pregnancy). The remaining 6 mothers had never smoked, which was verified through the cotinine analysis. Only 2 of the 20 mothers in the control group (10%) had a proven smoking habit. Information on cigarette smoking was also collected from fathers, with a positive match for 8 fathers in the SIDS group and 3 in the control group.

### Infant Death Circumstances

Circumstances of death, including place and position when the infants were put to sleep, were reported. Twenty-six newborns, including 24 SIDS and 2 controls, died during sleep. These infants were placed to sleep on their backs in the evening and found dead in the morning. They were not overdressed, and there were no soft pillows or other objects in sleep environment. The infant’s faces were not covered. The other 20 infants died during the day in various situations, mostly in the stroller or in the arms of their parents.

A summary of the main case profiles of this study is shown in Table 1. Ages are expressed in weeks as gestational age at birth, postnatal age, and postconceptional age at death.

**Table 1.** Main Profiles of the Study Cohort.

	SIDS		Controls	
Number of cases	26		20^a^	
Sex (male/female)	15/11		11/9	
Age (in weeks)	Range	*M* ± *SD*	Range	*M* ± *SD*
Postnatal age at death	4–30	14.57 ± 7.1^b^	8–29	15.5 ± 7.55
Gestational age at birth	26–41	35.38 ± 3.8^b^	27–41^c^	35.8 ± 3.39
Postconceptional age at death^d^	34–70	49.96 ± 8.6^b^	37–69	51.3 ± 8.7
Preterm birth	5^b^		4	
Dead during sleep	24^e^		2	
Position found at death (supine/prone)	16/10		14/6	
Maternal smoking (smokers/nonsmokers)	20/6^e^		2/18	
Paternal smoking (smokers/nonsmokers)	8/18^b^		3/17^f^	

*Note*. SIDS = sudden infant death syndrome.

a Autopsy diagnosis: congenital heart diseases (9), severe bronchopneumonia (4), pulmonary dysplasia (3), myocarditis (2), and malaria (1).

b Significance related to controls: ns (not significant).

c 2 missing values.

d Gestational age + postnatal age.

e Significance related to controls: *p* < .01.

f 3 missing values.

### Neuropathological Procedures

The anatomopathological protocol used for the in-depth study of the nervous system in cases of SIDS mainly included the examination of the brain stem, where the main centers controlling vital functions are located. The full methodology is available in our previous publications (Alfonsi and Crippa, 2016, Matturri et al., 2008; Roncati et al., 2017).

In this study, the examination was focused on the midbrain, where the SN is located. We dissected four or five tissue blocks from this portion of the brain stem, after fixation in 10% neutral-buffered formalin, at the root of the oculomotor nerve. The span of these samples exceeded the rostral and caudal extent of the SN.

All blocks containing SN were embedded in paraffin wax and then sectioned exhaustively with the microtome. Transverse serial 4-µm-thick sections were then made at intervals of 60 µm and processed according to the needs. Precisely, two of these sections were routinely stained with hematoxylin-eosin and Klüver-Barrera for the histological examination. Other sections were stained for histochemical and immunohistochemical investigations, as indicated later. Remaining sections were kept aside for further analyses, where appropriate.

The histological observations were performed with a Nikon Eclipse E800 light microscope (Nikon Corporation, Tokyo, Japan) equipped with an ocular micrometer, and images of interest were captured using a 40× objective lens and a Nikon Coolpix 8400 digital camera attached to the microscope, using the same settings and exposure times. More specifically, prior to image capturing, the camera was white balanced, and the exposure times were standardized to 0.055 ms.

#### Quantification of Neuron Density in SN

Klüver-Barrera-stained sections were also submitted to the densitometric analysis using an image analyzer (Image-Pro Plus, Media Cybernetics, Silver Spring, MD, USA). First, the outline of the two portions of the SN (the densely packed dorsal region, namely the SNpc, and the widespread ventral pars, the SNpr) in each of the two symmetrical parts was plotted. Then, images were acquired using a color video camera and displayed in a PC monitor. Only neurons with clearly defined boundaries were considered. The neuronal density of each of the two SN portions (SNpc and SNpr) was expressed as number of neurons per mm^2^. The overall data obtained from both SIDS and controls were reported as mean values and standard deviation (mean ± SD).

#### SN Neuron Counting

We applied a simple quantitative manual method to count the Klüver-Barrera-stained neurons of the SN in the same captured images for the densitometric evaluations, by using Adobe Photoshop’s counting tool (Adobe systems Inc., San Jose, CA, USA). We clicked for every case, in the same way as for the densitometric analysis, only the neurons which allowed for clear visualization of the nucleolus and precise definition of the cell walls, and then recording the number of cells.

#### TH Immunohistochemistry of the SNpc Neurons

TH, the rate-limiting enzyme in DA biosynthesis, was used as a dopaminergic neuronal marker. More specifically, for the immunohistochemical study of neuronal dopaminergic population within the SNpc, appropriate sections were incubated with rabbit anti-TH primary antibody (cat #AB152 Chemicon) diluted in phosphate buffered saline (PBS) (1:200) and reacted overnight at 4°C. Biotin-conjugated secondary antibody incubation (1:200 cat #S-1000 Vector Laboratories) was performed for 30 min at room temperature. After several washes in PBS, antibody complex was localized using the ABC system (Vectastain ABC Elite kit cat #PK6101, Vector Laboratories) followed by 3,3′-diaminobenzidine reaction. The sections were then counterstained with Mayer’s hematoxylin for nuclei and coverslipped after dehydration in ascending concentrations of ethanol and cleared in xylene. Negative controls were performed by preabsorbing the primary antibody with a relative antigen excess (100 g mL^–1^) and incubating the complex with the sections in the specific step, which always resulted negative.

##### Quantification of TH Immunohistochemical Results

To quantify the TH-immunopositive neurons in the SN, a qualitative rating system was applied to the two portions of the SN (SNpc and SNpr). A 4-point scale was used to quantify the percent of TH-immunopositive neurons in the delineated areas:

- 0 = *no TH immunopositivity*
- 1 = *immunopositivity in ≤20% of the neurons*
- 2 = *immunopositivity in >20% and ≤50% of the neurons*
- 3 = *immunopositivity in >50% of the neurons*

This qualitative TH-immunohistochemical analysis was complemented by the quantitative evaluation of immunopositive neurons with Adobe Photoshop’s counting tool, as described earlier.

#### NM Staining of the SNpc Neurons

The Schmorl’s reaction taken from Lillie’s method (Bancroft and Gamble, 2002) was applied to stain histological sections adjacent to those used for other examinations, as a reducing method for determining the presence of NM in the neurons. First, the sections were treated with a ferric-ferricyanide solution for 5 to 10 min and then washed under running tap water to ensure that all of the residual ferricyanide had been removed, after which a counterstain in Nuclear Fast Red Stain, Kernechtrot, was carried out for 5 min. Because melanin is able to reduce ferricyanide to ferrocyanide with the production of Prussian blue in presence of ferric salts, the NM under the microscope appeared dark blue in the neuron cytoplasms, while the nuclei were red-stained.

### Statistical Methods

All the histological, densitometric, and immunohistochemical findings, obtained according to the previously described procedures, were analyzed by two independent blinded pathologists. The evaluations obtained by each observer in relation to the different parameters were reported, case by case, in a table. Then, once the mean values were calculated, they were compared by using the K Index (KI) to evaluate the interobserver reproducibility. The Landis and Koch (1977) methodology for the interpretation of the K coefficient was then used, where 0 to 0.2 indicates slight agreement, 0.21 to 0.40 indicates fair agreement, 0.41 to 0.60 indicates moderate agreement, 0.61 to 0.80 indicates strong or substantial agreement, and 0.81 to 1.00 indicates very strong or almost perfect agreement (a value of 1.0 implying perfect agreement). Very satisfactory KI values (from 0.86 for densitometric evaluation to 0.88 for histological ones) were obtained in this study. The statistical significance of the direct comparisons between groups was determined by analysis of variance followed by a Tukey’s post hoc test. Statistical calculations were carried out using the SPSS (statistical package for social science) statistical software. Differences were considered statistically significant if the *p* value was <.05. Data are expressed as mean ± standard deviation or numbers with the corresponding percentages.

## Results

### Morphological Examination of the SN

**Figure 1.**
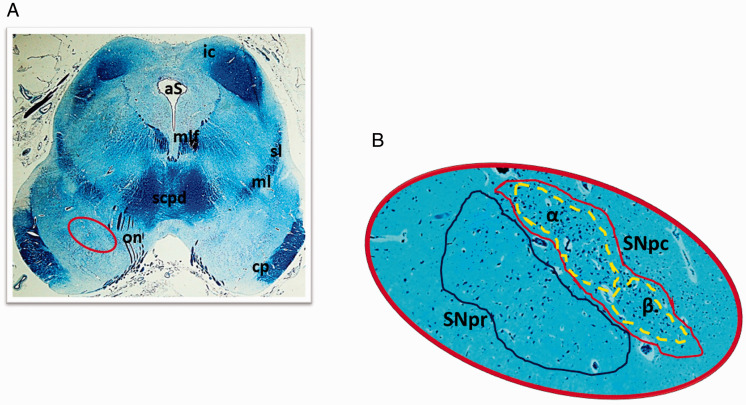
Substantia Nigra. (A) Photomicrographs of a transverse section of midbrain, at the level of inferior colliculus. The circled area, shown at great magnification in (B), indicates the different portions of the Substantia Nigra. Klüver-Barrera stain. Magnification: (A) 0.5×; (B) 10×. aS = aqueduct of Sylvius; cp = cerebral peduncle; ml = medial lemniscus; mlf = medial longitudinal fasciculus; on = oculomotor nerve; ic = inferior colliculus; scpd = superior cerebellar peduncle decussation; sl = spinal lemniscus; SNpc = substantia nigra pars compacta; SNpr =substantia nigra pars reticulata; α = subnucleus α of the pars compacta; β = subnucleus β of the pars compacta.

**Figure 2.**
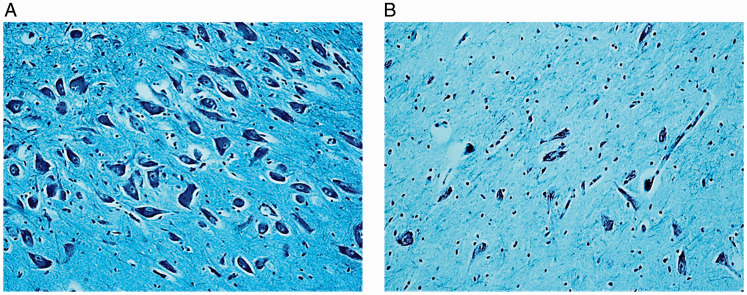
Substantia Nigra Pars Compacta (SNpc). Comparison between the neuron density in a control case (A) and in a case of SIDS (B). Klüver-Barrera stain. Magnification: (A and B) 20×.

The histological examination of the SN, the main focus of this study, was performed on transversal serial sections of the caudal midbrain preferably stained with Klüver-Barrera method. In all the control cases, this structure resulted located adjacent to the superior peduncles, with a bilaterally symmetrical “strip-like” shape. Figure 1 highlights the localization of the SN and its components according to the human brain stem atlas of Büttner-Ennever and Horn (2014), that is, the SNpc, with a densely packed and numerous population of neurons, subdivided into subnucleus α, and subnucleus β, less extensive and with a more rarefied pattern of neurons than that of subnucleus α, and the SNpr, a relatively poor cell structure lying ventral to the SNpc, much larger and more ovoid in shape than the SNpc. The soma of all the neurons are polygonal or fusiform in shape with a light, frequently eccentric nucleus, evident nucleolus, and abundant cytoplasm. The dendrites and axons are well visible and intertwined with each other especially in the SNpc (Figure 2A).

**Figure 3.**
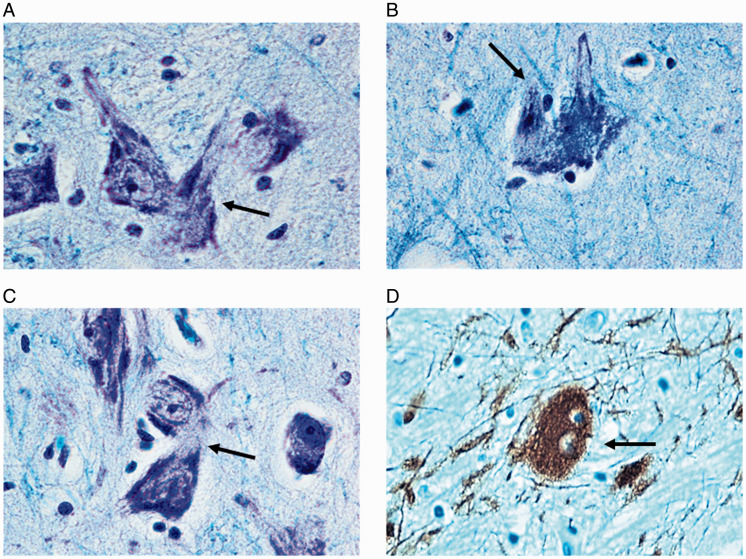
Mitotic neurons in the Substantia Nigra. A panel of photomicrographs showing terminal mitotic figures (highlighted by arrows in A, B, and C) and a binucleate neuron (D) found in the SNpc. (A to C) Klüver-Barrera stain; (C) *Thyrosine hydroxylase* immunohistochemistry. Magnification: 100×.

 Hypoplasia of the SN mainly affecting the SNpc was observed in 18 SIDS cases aged 8 to 21 postnatal weeks. Two of these were born preterm at 27 and 29 gestational weeks and died at 14 and 15 postnatal weeks, respectively. Hypoplasia was defined by comparing the histological pattern of the SN in these 18 SIDS with that of age-matched controls, especially taking into account the gestational age at delivery. In particular, the finding of the two preterm cases were compared with those of two of the four premature cases of the control group with similar pre- and postnatal age (28 and 29 weeks at delivery, respectively; 14 postnatal weeks at death for both).The hypoplasia in all case was characterized by a greatly reduced number of neurons with rare processes, compared with those of the control group (Figure 2B). These observations were validated by densitometric evaluations, as reported later. Noteworthy was the frequent presence of mitotic figures (telophases) and binucleate neurons in the SNpc of SIDS cases (Figure 3). This finding was rarely detected in controls.

### Neuron Density

Overall, the SIDS cases had a significantly lower neuron density in the SNpc compared with the controls (*p* < .01). The mean values in SNpc were 36.5 ± 32.7/mm^2^ for the SIDS group and 92.8 ± 15.3/mm^2^ for the control group. The number of neurons per mm^2^ in SNpr was almost overlapping in the two groups (5.44 ± 2.43 in SIDS and 5.52 ± 3.1 in controls). Table 2 summarizes the densitometric evaluations.

**Table 2.** Analysis of the SN Neuronal Density (Cells/mm^2^) in SIDS and Controls.

	SIDS	Controls
SNpc		
Range	5–100	72–120
Mean value ± SD	36.5 ± 32.7*	92.8 ± 15.3
SNpr		
Range	1–9	2–10
Mean value ± *SD*	5.44 ± 2.43	5.52 ± 3.1

*Note*. SIDS = sudden infant death syndrome; SNpc = substantia nigra, pars compacta; SNpr = substantia nigra, pars reticulata; SD = standard deviation.

* Significance related to controls: *p* < .01.

### Neuron Counting

We find a wide range of total neuron number from 287 to 535 for controls (mean: 396.9) and a much lower number (from 70 to 320; mean: 152.3) for SIDS.

### TH Immunohistochemistry

**Figure 4.**
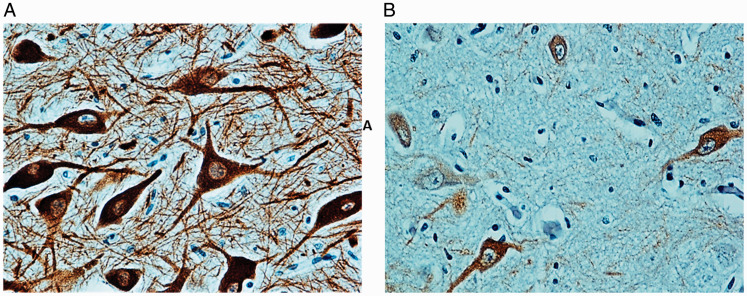
*Thyrosine Hydroxylase* (TH) Immunohistochemistry. (A) Intense immunopositivity in the neuronal bodies and processes in the SNpc of a control cases. (B) Rare presence of weakly immunostained neurons in the SNpc of a SIDS case. Magnification: 40×.

**Figure 5.**
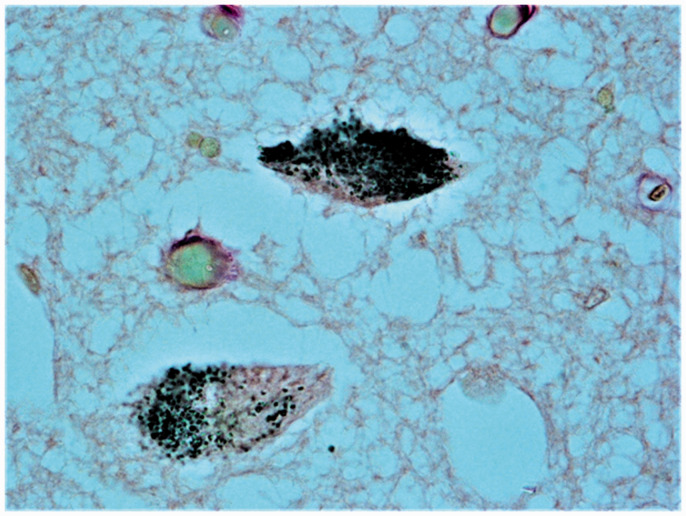
Neuromelanin (NM) Histochemistry. Pigmented neurons in the SNpc of a control infant. Magnification: 40×.

The percentage of TH-immunopositive neurons in the control group ranged from 45% to 90% (scores 2–3) in the SNpc (Figure 4A). Contrastingly, a greater loss of TH immunopositivity in the SNpc neurons was observed prevalently in SIDS victims compared with controls (Figure 5B). In fact, the immunopositivity in SNpc was classified as 0 or 1 (with ≤20% of TH-immunopositive neurons) in 22 of the 26 SIDS cases (85%) and only in two controls (10%). Only a few positive neurons were occasionally found in the SNpr of both the controls and the SIDS cases, which probably migrated from the adjacent SNpc.

The total number of TH-immunopositive neurons ranged from 189 to 288 with a mean value of 244.4 in the control cases. Much lower numbers of TH-immunostained neurons, precisely from 9 to 180 (mean value: 39.5), were detected in SIDS group.

### Analysis of Data in SIDS and Controls Divided Into Age-Matched Groups

A more detailed analysis of the results presented so far was then performed dividing the cases, both controls and SIDS, into six groups according to age, as shown in Tables 3 and 4. 

**Table 3.** Neuronal Density, Total Neuron Number, TH Immunopositivity, and Histology of the SNpc in Control Cases Divided in Groups According to Age.

Groups	Age (in weeks)	Number of cases	Neuron density		Neuron number		TH-immunopositive neuron number		Histology	
			Range	*M*	Range	*M*	Range	*M*	Normal	Hypoplasia
I	4–8	4	101–120	108	492–535	506.5	221–246	232.2	4	/
II	9–12	2	100–120	110	521–530	525.5	234–265	249.5	2	/
III	13–16	3	89–100	92.6	328–403	363.6	213–240	230.3	3	/
IV	17–20	6	85–97	90.5	333–362	344.8	189–288	234.1	6	/
V	21–24	3	72–79	76.6	320–361	340.3	210–288	256.6	3	/
VI	25–30	2	68–71	69.5	287–315	301	252–275	263.5	2	/

*Note*. *M* = mean; TH = tyrosine hydroxylase.

**Table 4.** Neuronal Density, Total Neuron Number, TH Immunohistochemistry, and Histology of the SNpc in SIDS Cases Divided in Groups According to Age.

Groups	Age (in weeks)	Number of cases	Neuron density		Neuron number		TH-immunopositive neuron number		Histology	
			Range	*M*	Range	*M*	Range	*M*	Normal	Hypoplasia
I	4–8	5	8–20	16.8	89–320	142	17–180	109.0	2	2
II	9–12	5	13–29	17	83–104	96	16–78	29.6	1	4
III	13–16	7	5–20	13.6	98–298	144.3	17–55	30.4	/	7
IV	17–20	3	23–88	58.3	70–99	85.6	9–18	14.0	/	3
V	21–24	3	59–89	74.3	75–298	194.3	9–27	16.6	1	2
VI	25–30	3	88–100	95.6	189–316	251.6	15–82	38.3	3	/

*Note*. *M* = mean; TH = tyrosine hydroxylase.

The data analysis in Table 3 related to the control cases in which the SNpc showed a normal structure, highlighted a slight increase in the mean number of neurons and neuronal density in the first 3 months (I and II groups/4 to 12 postnatal weeks), followed by a progressive decrease in the past 4 months (III to VI groups; 13–30 weeks). The number of TH-immunopositive neurons in the various age groups on the contrary did not change remarkably. However, to a careful microscopic observation, these immunostained neurons showed a progressive increase of their size and branching with the age. A well evident decrease in neuron density and TH-immunopositivity was detected in the SIDS age groups compared to controls (Table 4).

### Neuromelanin

In four infants over 24 postnatal weeks (6 months) of age belonging to the control group, dark cytoplasmic granules were detected in several neurons of the SNpc, which was indicative of NM content (Figure 5). Conversely, NM was not found in the SIDS cases, even in the three older victims, over 24 postnatal weeks of age.

### Correlations of Results With Maternal Smoking

All of the results were associated with maternal prenatal smoking. A significant correlation was observed between SIDS, SNpc hypoplasia, SNpc negativity/low TH immunostaining, and nicotine absorption in utero. In particular, infants who died of SIDS were more likely to be exposed to maternal cigarette smoke than controls (20 SIDS and 2 controls); 17 of these 20 SIDS victims and 1 of the 2 control subjects with smoker mothers showed TH scores = 0 or 1 in the SN neurons. Conversely, low TH expression was detected in 7 of the 24 victims with nonsmoker mothers. Association between SNpc hypoplasia and maternal smoking was detected exclusively in 15 SIDS. Table 5 shows these correlations.

**Table 5.** Correlation of Results With Maternal Smoking in Pregnancy.

Categories	Maternal smoking		*p* value
	Yes^a^ (n.22)	No (n.24)	
Cases			
SIDS (n.26)	20 (77%)	6 (23%)	<.01
Controls (n.20)	2 (10%)	18 (90%)	
SNCpc cytoarchitecture^b^			
Hypoplasia (n.18^c^)	15 (83%)	3 (17%)	<.05
Normal (n.28)	7 (25%)	21 (75%)	
TH immunoexpression			
Score 0–1 (n.24)	17 (71%)	7 (29%)	<.01
Score 2–3 (n.22)	5 (23%)	17 (77%)	

*Note*. SIDS = sudden infant death syndrome; SNpc = substantia nigra, pars compacta; TH = tyrosine hydroxylase.

a Smoking 3 to 10 cigarettes a day.

b Validated by densitometric and quantitative analyses.

c All belonging to the SIDS group.

**Figure 6.**
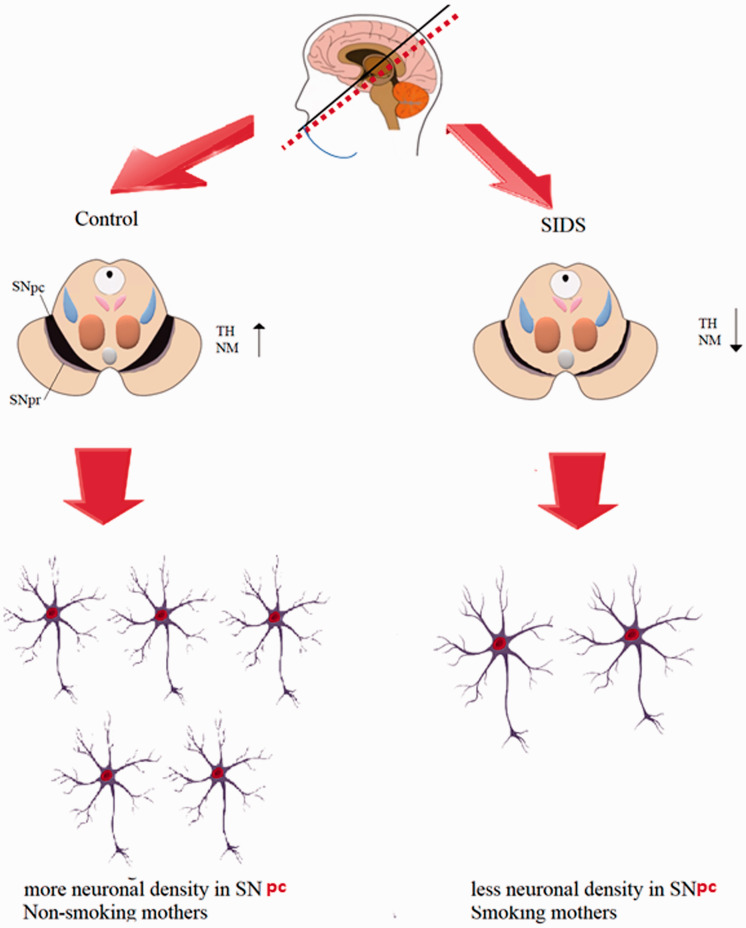
Schematic Representation of All the Results Obtained in This Study. It is evident that the SN in SIDS cases is characterized by a decrease in the neuron number and in the expressivity of both the TH enzyme and NM with respect to the control cases and furthermore that these results are closely related to the exposure to cigarette smoke. SIDS = sudden infant death syndrome; TH = tyrosine hydroxylase; NM = neuromelanin; SNpc = substantia nigra, pars compacta; SNpr = substantia nigra, pars reticulata.

Finally, Figure 6 summarizes schematically all the results obtained in this study.

## Discussion

To the best of our knowledge, this study provides the first direct evidence of SN morphological alterations in SIDS. In particular, because the SN, and precisely the SNpc, is known to be a major DA-producing region of the brain, the loss of DA neurons from the SNpc provides us with valuable insight into the pathogenetic mechanism of this syndrome. It is well known that among its various functions, DA regulates the sleep–wake cycle (Dzirasa et al., 2006), and in most cases, SIDS occurs during arousal from sleep (Hunt, 1989; Kahn et al., 2003; Kato et al., 2003).

Much attention has been paid to the involvement of DA in locomotor activity, cognitive functions, and sensorimotor integration (Nieoullon, 2002; Nieoullon and Coquerel, 2003; Bissonette and Roesch, 2016). The degeneration of dopaminergic neurons within the SNpc has long been known to be a hallmark feature of PD, a neurodegenerative disorder mainly characterized by motor deficits and cognitive impairment (as executive function deficits, attention and learning difficulties, etc.; Brooks, 1997; Jankovic, 2008). Further studies have highlighted the role of DA in additional physiological functions such as ventilation and neurological modulation of sleep (Kolesnikova and Serebrovskaya, 1998; Cabezas et al., 2003; Dzirasa et al., 2006; Lima et al., 2007). In particular, during the arousal phase from sleep to wakefulness, there is an increase in the firing activity of dopaminergic neurons of the SNpc (Dzirasa et al., 2006; Lima et al., 2007, 2009). The pressing need to increase DA synthesis is justified by the fact that physiological arousal requires a radical change in brain activity, as it is characterized by hyperventilation, greater motor activity, and increased responsivity to sensory and emotional inputs (Berry and Gleeson, 1997; Jones, 2003). A sudden arousal from sleep is also required as a life-saving reflex in case of hypoxia and/or hypercapnia (due to accidental airway obstruction, severe bronchial disease, prone sleeping position, nicotine absorption, etc.; McGinty and Szymusiak, 2000).

Defects in sleep arousal in response to a life-threatening stressor is deemed to be the main cause of SIDS (Hunt, 1989; Kato et al., 2003). Sudden infant death is primarily attributed to respiratory failure caused by physiological increase of the respiratory rate that usually occurs under hypoxic conditions to regularize the plasmatic gas values (Kahn et al., 2003).

The results of our study indicate that the SN, and especially the SNpc, undergoes delayed development in most SIDS victims with a reduced number of neurons sometimes characterized by immature features (hypoplasia). These observations were deduced by comparing SIDS and controls of the same age. In control subjects, in which the SN showed a regular pattern, after a slight increase in the mean number of neurons of the SNpc in the first 3 months, a progressive decrease, plausibly apoptotic in nature (Ries et al., 2009), was observed in the subsequent stages of maturation. This developmental model recalls the one observed in an experimental study by Bandeira et al. (2009). These authors demonstrated that postnatal rat brain growth proceeds in different phases, at first related to the addition then to the elimination of neurons by cell death.

In addition, on using the rate-limiting enzyme TH as a marker for dopaminergic neurons, a significant loss of TH-immunopositive neurons was observed in SIDS, both in cases with normal or reduced neuronal density, compared with the controls. Our results contrast with those reported by Obonai et al. (1998). These authors, in a study on the catecholamine neurons in the brain stem of SIDS victims, reported that TH immunoreactivity was definitely decreased in adrenergic and noradrenergic neurons of the vagal nuclei, locus coeruleus, and area reticularis superficialis ventrolateralis and slightly reduced but not in a significant way in dopaminergic neurons of the SN.

In support to our statements, electrophysiological data indicated that the loss of only half of the dopaminergic neurons within the SNpc leads to a significant impairment in sleep–wake parameters (Lima et al., 2007). We can therefore hypothesize that the deficit of dopaminergic neurons by us demonstrated in the SNpc of SIDS victims, given the important role played by the DA in regulation of the sleep–wake cycle, may have contributed to fatal outcomes, considering that the majority of the SIDS deaths occurred upon awakening from sleep. The DA deficit may also have hindered the essential motor response of the newborn to a life-threatening stressor which reduces the amount of oxygen supplied to the brain. There is strong evidence that maternal smoking during pregnancy is a major risk factor for SIDS. This study is further confirmation that nicotine absorption in uterus may have contributed to death from SIDS causing, in addition to the various damages already known, also a significant decrease of neuron density in the SN. The hypoplasia of the SN, characterized by a greatly reduced dopaminergic neuron number, was in fact found in 83% of SIDS cases with a smoking mother and only in 17% of those with proven negative nicotine absorption. It is known that nicotine, if aspirated from the pregnant mother, by crossing the placental barrier, can be rapidly detected in the fetal bloodstream at levels exceeding maternal concentrations by 15% (Wickström, 2007). Consequently, the nicotine, being one of the few lipid-soluble substances capable of overcoming the blood–brain barrier by passive diffusion, can impact the brain development. Accordingly, experimental studies have shown that in utero exposure to maternal smoking is associated with neuronal mitotic abnormalities and increased programmed cell death with consequent reduction of the neuronal content (Roy et al., 1998; Chatterton et al., 2017). Moreover, our previous SIDS studies revealed a close correlation between maternal smoking during pregnancy and defects in the morphological/functional development of various nervous system structures (Lavezzi et al., 2010, 2012, 2013b, 2013c, 2017; Lavezzi, 2018). This study enhances our knowledge on the devastating effect of cigarette smoking and reinforces our awareness that once nicotine crosses the blood–brain barrier, it can harm and deactivate important brain centers which are crucial to perform the vital activities.

But how could the prenatal exposure to nicotine have determined a decrease of the neuron number in SN of the fetal brain? We hypothesize that nicotine is able to perturb the normal brain maturation also by impairing the central cholinergic system through incorrect regulation of nicotinic acetylcholine receptor (nAChR) binding. Because different subunits of nAChRs (precisely alpha 4 and alpha 6 subunits) have been demonstrated in the dopaminergic neurons of the SN (Göldner et al., 1997; Arroyo-Jim nez et al., 1999), we can assume that alterations of these receptors caused by nicotine may contribute, as well as alterations of the dopaminergic transmission, to alter the neuronal component of the SN. This hypothesis is supported by experimental studies showing that stimulation of nAChRs by nicotine causes neuronal inhibition of DNA synthesis, mitotic abnormalities, and apoptosis and ultimately a deficit in the total number of neurons in cholinergic structures (Slotkin, 2004).

It is important to note the differences in neuronal density observed in the SNpc of newborns compared with the neuronal density of adult subjects. Our results indicate a mean neuronal density in the control infants of 92.8 ± 15.3/mm^2^, which is definitely a higher value than that observed in adult humans, as reported in literature (Ross et al., 2004; Abbott et al., 2016; Robert et al., 2017). These studies in fact, carried out on the SN albeit on different topics, reported mean values of approximately 20 neurons per mm^2^. It is well known that the decline in the number of adult SNpc neurons may be due to a progressive loss of neurons, which is an inevitable consequence of normal aging (Koukolík, 1989; Brody, 1992; Uylings et al., 2000 ) and that the numeric atrophy through age affects mainly the noradrenergic locus coeruleus and the dopaminergic SN (Manaye et al., 1995; Robert et al., 2017).

Another noteworthy finding in this study concerns the NM, a dark-stained pigment which is usually produced and stored in intracellular granules in specific populations of catecholaminergic neurons of the human brain such as the locus coeruleus and especially the SNpc (Fenichel and Bazelon, 1968; Zecca et al., 2001). Histochemical studies have demonstrated that this dark pigment in the human brain has similar properties to the melanin found in skin, hair, and eyes, being insoluble to organic solvents and markable with silver stains (Nicolaus, 2005; Plum et al., 2016), which was why it was called *neuromelanin.* However, little is known about the biology and the physiological functions of NM. We only know that it is directly biosynthesized from levo-dopa precursor in DA synthesis (Zecca et al., 2001). A much debated issue is the involvement of NM in cell iron homeostasis and in the neuroprotective function by sequestration of toxic substances (Aime et al., 1997; Bridelli et al., 1999; Bolzoni et al., 2002; Zucca et al., 2017).

In this study, it was observed that the NM in controls is normally unexpressed in the SN in the first months of life and only begins to appear in the form of cytoplasmic dark granules in several neurons of the SNpc from 6 months of age (24 postnatal weeks) onward. This result is inconsistent with current knowledge that NM pigment in the SN is not visible under light microscopy in newborns and becomes visible only at 2 to 3 years of age (Fenichel and Bazelon, 1968). However, it is interesting to note that, as reported in one of our previous studies (Lavezzi et al., 2013a), the NM in the locus coeruleus can already be identified in 2- to 3-month-old infants. As yet we are unable to explain these differences in synthesis times. In this study, NM was not detected in the SIDS cases, even in the victims over 6 months of age. It is also worth mentioning that the NM deficiency was understandably related to the decrease in TH, as both NM and TH are by-products of tyrosine.

Also the observation concerning the frequent presence of mitotic/binucleated neurons in SIDS cases and only rarely in controls is to be reported. The pathogenetic mechanism for their formation is still unknown. It is well known that during prenatal nervous system differentiation, neuroblasts undergo mitotic activity and migrate to reach their definitive location, where they differentiate into mature neurons that are characterized by the permanent exit from the cell cycle (Marin and Rubenstein, 2003; Ayala et al. 2007; Cayre et al., 2009). However, according to various authors, if necessary, neurons can reactivate the molecular system which is capable of promoting cell proliferation which was previously blocked (Zang, 1989; McShea et al., 1999; Arendt, 2003; Zhu et al., 2008). The activated neurons in the SNpc detected in this study could represent an attempt to proliferate to promote a hyperplastic process as a reaction to noxious stimuli and unfavorable circumstances. The functional significance of these neurons obviously merits further investigation.

In conclusion, although the mechanisms resulting in SIDS are still unknown, this study offers a contribution to the understanding of the pathogenesis of this syndrome, particularly in cases, which represent the majority, occurring during sleep. Here, we highlighted in fact for the first time the important role of the SNpc in SIDS and stressed once again, according to our previous research (Lavezzi et al., 2010, 2012, 2013b, 2013c, 2017; Lavezzi, 2018), that cigarette smoke absorption may have strongly influenced the results presented here.
